# Service organisation for people with dementia after an injurious fall: challenges and opportunities

**DOI:** 10.1093/ageing/afz010

**Published:** 2019-03-28

**Authors:** Alison Wheatley, Claire Bamford, Caroline Shaw, Miriam Boyles, Chris Fox, Louise Allan

**Affiliations:** 1 Institute of Health and Society, Newcastle University, Newcastle upon Tyne, UK; 2 Norwich Medical School, University of East Anglia, Norfolk, UK; 3 Institute of Neuroscience, Newcastle University, Newcastle upon Tyne, UK; 4 Institute of Health Research, University of Exeter, Exeter, UK

**Keywords:** dementia, falls, care pathways, older people

## Abstract

**Introduction:**

people with dementia are more likely to fall and less likely to recover well after a fall than cognitively intact older people. Little is known about how best to deliver services to this patient group. This paper explored current service provision to help inform the development of a new intervention.

**Methods:**

qualitative approaches were used to explore the views and experiences of people with dementia, family carers and professionals providing services to people with dementia following an injurious fall. These data were analysed using a thematic, iterative analysis.

**Findings:**

while a wide range of services potentially relevant to people with dementia was identified, there were no dedicated services for people with dementia with fall-related injuries in our three geographical areas. Factors influencing service uptake included a lack of knowledge of local provision amongst professionals and underdeveloped information sharing systems. Some aspects of current service organisation were incompatible with the needs of people with dementia. These include an emphasis on time-limited interventions; lack of longer-term follow-up; and service delivery in environments that could be challenging for people with dementia.

**Conclusions:**

care pathways for people with dementia who fall are fragmented and unclear. This is likely to preclude people with dementia from receiving all appropriate support and contribute to poor recovery following a fall. The findings highlight the need for new approaches to service organisation and delivery which address the specific needs of people with dementia who fall.

## Key points

- People with dementia living in the community are at risk of falls, the consequences of which may include physical injury, loss of conditioning, and fear of further falls.
- Current care pathways for people with dementia who fall are often fragmented and unclear.
- Organising services in ways that compensate for the symptoms of dementia and cognitive impairment may facilitate recovery.

## Introduction

More than a third of people living in the community over the age of 65 fall each year, rising to half of those over 80 [1]. In addition to physical consequences of falls, fallers may also experience ‘fear of falling’ which can result in loss of conditioning and increased risk of future falls [2]. Treating falls in older people has an additional economic burden [3]. These consequences have led to an international emphasis on reducing and preventing falls [4].

For older people with dementia, the risk of falls is more than double that of cognitively intact older people [5] and between 60% and 80% of people with dementia will have at least one fall in a 12-month period [6, 7]. Evidence shows people with dementia are less likely to recover well after a fall and more likely to require increased care than other older adults [8]. Preventing falls and maximising recovery after a fall is therefore crucial to achieve the overarching goal of the National Dementia Strategy for England of ‘living well with dementia’ [9].

Evidence on how best to prevent and manage falls in people with dementia is limited and difficult to synthesise; reviews have been unable to draw definitive conclusions or to make clear recommendations for clinical practice [10–12]. New UK dementia guidelines recommend that falls services address the specific needs of people with dementia [13]. It is, however, unclear how this can be achieved in practice. There is therefore a need to research the most appropriate methods of caring for people with dementia following a fall.

To inform the content and delivery of a new intervention, we critically examined the range of services currently available, and elicited stakeholder views on existing services and suggestions for improvements. The data reported in this paper are drawn from the same cohort as a previously published report [14], which focused specifically on staff training needs and the impact of individual staff skill on the outcomes for people with dementia. In contrast, the current paper explores structural barriers and facilitators to successful care for people with dementia following a fall.

## Methods

We used interviews and focus groups to explore the views and experiences of people with dementia who had experienced a fall, family carers and health and social care professionals. We supplemented these data with direct observation of care to provide insight into how falls injuries in people with dementia are currently managed. Details of the methods used have been reported in full elsewhere [14].

For the initial interviews and focus groups with professionals, ethical review was provided by Newcastle University and any necessary permissions obtained from research and development departments of participating Trusts. Approval for observation and interviews with patients and carers was given by Newcastle and North Tyneside 1 Ethics Committee (reference 15/NE/0397); Newcastle and North Tyneside 2 Ethics Committee (reference 16/NE/0011); and the Health Research Authority. Additional approvals were received from participating Trusts and Social Services Departments as required. For non-statutory agencies, approval was sought from senior managers.

## Results

### Participants

We interviewed 53 health and social care professionals, 8 patients and 9 carers. A further 28 professionals took part in five focus groups. We observed 20 professionals delivering care or instruction to 85 patients/clients in a range of settings, including hospitals and patients’ homes.

### Findings

Although we identified a range of services used by people with dementia with fall-related injuries, a number of shortcomings limited their utility. These included the lack of:

- clear pathways for people with dementia with fall-related injuries
- information sharing and poor communication between services
- continuity of care and ongoing interventions.

Each of these is discussed below.

#### No clear pathway for people with dementia with fall-related injuries

No specific services for people with dementia were identified at any site. Moreover, people with dementia were sometimes excluded from services that were offered to other older people, particularly if their falls were attributed to dementia:

I think if it’s purely that, it’s their behaviour because of the dementia, [causing the falls] then in some respects, they won’t necessarily benefit from the falls clinic, because they don’t need all the other investigations.

**(Interview, Prof 53, physiotherapist, outpatient falls service)**

**Figure 1:**
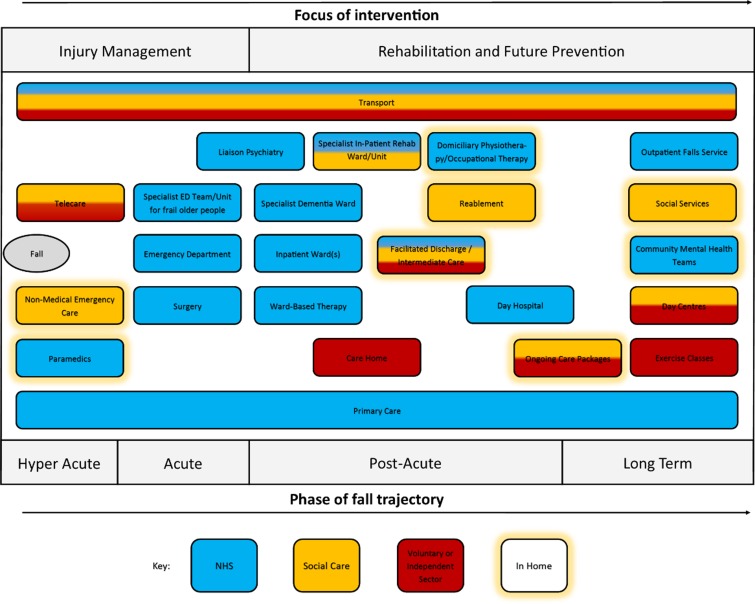
Overview of services potentially available to people with dementia with fall-related injuries

We identified 21 distinct service ‘types’ to which people with dementia with a fall-related injury could be referred. Figure 1 shows a composite set of these services (as not all service types were available in all sites). The horizontal position of services indicates the point in the falls trajectory at which they are typically involved (hyper acute, acute, post-acute or long term) and the focus of the intervention provided (either injury management or rehabilitation and prevention of future falls). The colour(s) indicate the typical service provider(s) and a yellow halo indicates that a service is home-based. While the pathway might appear linear, it is possible for many of these services to be accessed in parallel and people with recurrent falls may have multiple iterations of different service configurations. It is therefore difficult to identify a ‘typical’ patient pathway.

Few professionals were aware of the full range of services available. Paramedics found it particularly difficult to keep up with the complexity and turnover of services across NHS Trust boundaries; they suggested a single point of contact system similar to that used for stroke. Mapping or developing pathways in each locality would enable existing resources to be used appropriately. Ensuring that information, particularly inclusion/exclusion criteria, remained up to date was stressed; possible strategies were to appoint a ‘link person’ or produce an app.

#### Information sharing and communication between services

At an organisational level, responsibility for falls and dementia was typically allocated to different individuals. Where there was a combined falls and dementia lead, staff were usefully able to seek advice or request joint working when dealing with challenging situations. Lack of joint working could create problems, particularly if conflicting interventions were suggested:

[Home carers] changed some of the equipment over because they thought it wasn’t suitable, even though the hospital had provided what they thought was right. […] Although that [hoist] is very useful, the stand assist they brought originally meant Dad had to work at it, which exercised his arm muscles and his leg muscles and that’s what I would have preferred.

**(Interview, Patient 8 and Carer 8)**

Joint information systems remained underdeveloped, with many services unable to access even basic information from others, including dementia diagnoses. Multidisciplinary team meetings offered one solution.

#### Continuity of care and on-going interventions

Continuity of care was valued by all participants. However, existing services were often limited by time or funding, which created discontinuity. Many professionals argued that people with dementia needed a longer intervention than other older people. Flexibility in duration of support, with session length and frequency tailored to the individual, was stressed, including time to get to know the person:

I think the time aspect is the main thing, because sometimes you’ve just got to figure out, for that patient, what’s going to work. And sometimes that can take a few sessions really, before you actually get into the actual rehab bit.

**(Focus group,Prof 80**, **clinical lead physiotherapist, community mental health team and specialist dementia unit)**

On-going follow-up was seen as important to sustain functional improvements and ensure that existing equipment was still appropriate to the changing needs of people with dementia. Opportunities for review and maintenance activities were currently limited; patients were typically discharged to primary care where they would not necessarily be regularly reviewed.

## Discussion

Our data suggest that care pathways for people with dementia who fall are often fragmented and unclear. There is evidence, though not specific to dementia and falls, to suggest that formalised care pathways may provide advantages such as reduced time between diagnosis and treatment, greater consistency of care, reduced costs, and improved patient outcomes [15–18]. A coherent pathway for people with dementia after a fall may therefore be beneficial, particularly if there is a focus on collaborative working across the boundaries of health, social care and third-sector provision. However, the integration of health and social care services in the UK remains challenging despite numerous initiatives to improve information sharing and communication between services [19]. Barriers to implementation may occur at the staff, organisational or financial level and may include lack of knowledge, negative attitudes towards guidelines or pathways, lack of training in the use of pathways and increased costs [20]. Despite evidence that continuity is highly valued by people with dementia and carers [21], existing services tend to be time-limited interventions which can create discontinuity of care; [22, 23] more evidence is needed to clarify the optimal duration of interventions and the extent to which this should be tailored to individual people with dementia. There is evidence to support a ‘patient-centred’ approach to rehabilitation of people with dementia following hip fracture [24]. Further research is needed to determine its applicability to the broader question of care for people with dementia with fall-related injuries.

### Recommendations for practice

Based on this work, we recommend:

- developing local and/or national falls pathways specifically for people with dementia which include services provided by health care, social care and the third-sector
- increasing the flexibility of the structure and content of existing reablement and rehabilitation services to better meet the needs of people with dementia.

The findings of this qualitative work were used to inform the development of a new intervention for people with dementia following a fall [25].

## Notes

**Acknowledgements:** We are grateful to all of the participants, to Beth Edgar for administrative support, and to members of the wider DIFRID project team.

**Declaration of Conflict of Interest:** None.

**Declaration of Sources of Funding:** This work was supported by the National Institute for Health Research Health Technology Appraisal [grant number 13/78/02]. The views expressed are those of the authors and not necessarily those of the NHS, the NIHR or the Department of Health.

## References

[1] Tinetti ME, Speechley M, Ginter SF Risk factors for falls among elderly persons living in the community. N Engl J Med 1988; 319: 1701–7. 3205267 10.1056/NEJM198812293192604

[2] Jorstad EC, Hauer K, Becker C, Lamb SE, ProFa NEG Measuring the psychological outcomes of falling: a systematic review. J Am Geriatr Soc 2005; 53: 501–10. 15743297 10.1111/j.1532-5415.2005.53172.x

[3] Heinrich S, Rapp K, Rissmann U, Becker C, Konig HH Cost of falls in old age: a systematic review. Osteoporos Int 2010; 21: 891–902. 19924496 10.1007/s00198-009-1100-1

[4] World Health Organisation WHOQOL-OLD Manual. European Office, Copenhagen: 2006.

[5] Muir SW, Gopaul K, Montero Odasso MM The role of cognitive impairment in fall risk among older adults: a systematic review and meta-analysis. Age Ageing 2012; 41: 299–308. 22374645 10.1093/ageing/afs012

[6] Allan LM, Ballard CG, Rowan EN, Kenny RA Incidence and prediction of falls in dementia: a prospective study in older people. PLoS One 2009; 4: e5521. [Electronic Resource]. [Controlled Clinical Trial Research Support, Non-U.S. Gov’t]. 19436724 10.1371/journal.pone.0005521 PMC2677107

[7] Shaw FE Prevention of falls in older people with dementia. J Neural Transm (Vienna) 2007; 114: 1259–64. 17557130 10.1007/s00702-007-0741-5

[8] Shaw FE Falls in cognitive impairment and dementia. Clin Geriatr Med 2002; 18: 159–73. [Review]. 12180241 10.1016/s0749-0690(02)00003-4

[9] Department of Health Living with Dementia: A National Dementia Strategy. London: Department of Health, 2009 Available at https://www.gov.uk/government/publications/living-well-with-dementia-a-national-dementia-strategy (20 January 2012, date last accessed).

[10] Robalino S, Nyakang’o SB, Beyer FR, Fox C, Allan LM Effectiveness of interventions aimed at improving physical and psychological outcomes of fall-related injuries in people with dementia: a narrative systematic review. Syst Rev 2018; 7: 31. 29463292 10.1186/s13643-018-0697-6 PMC5819703

[11] Booth V, Hood V, Kearney F Interventions incorporating physical and cognitive elements to reduce falls risk in cognitively impaired older adults: a systematic review. JBI Database System Rev Implement Rep 2016; 14: 110–35. 10.11124/JBISRIR-2016-002499 27532469

[12] Smith T, Hameed Y, Cross J, Sahota O, Fox C Assessment of people with cognitive impairment and hip fracture: a systematic review and meta-analysis. Arch Gerontol Geriatr 2013; 57: 117–26. [Meta-Analysis Review]. 23680535 10.1016/j.archger.2013.04.009

[13] National Institute for Health and Care Excellence Dementia—assessment, management and support for people living with dementia and their carers. NICE; 2018 Available at https://www.nice.org.uk/guidance/ng97 (7 February 2019, date last accessed). 30011160

[14] Bamford C, Wheatley A, Shaw C, Allan L Equipping staff with the skills to maximise recovery of people with dementia after an injurious fall. Aging Mental Health 2018: 1–9. 10.1080/13607863.2018.1501664. 30428699

[15] Schrijvers G, van Hoorn A, Huiskes N The care pathway: concepts and theories: an introduction. Int J Integr Care 2012; 12: 1–7. 10.5334/ijic.812 PMC3602959 23593066

[16] Deneckere S, Euwema M, Van Herck P et al Care pathways lead to better teamwork: results of a systematic review. Soc Sci Med 2012; 75: 264–8. 22560883 10.1016/j.socscimed.2012.02.060

[17] Allen D, Gillen E, Rixson L Systematic review of the effectiveness of integrated care pathways: what works, for whom, in which circumstances? Int J Evid Based Healthc 2009; 7: 61–74. 21631848 10.1111/j.1744-1609.2009.00127.x

[18] Centre for Policy on Ageing The Effectiveness of Care Pathways in Health and Social Care: Rapid Review. London: CPA, 2014.

[19] Morse A Health and Social Care Integration. London: National Audit Offic, 2017.

[20] Evans-Lacko S, Jarrett M, McCrone P, Thornicroft G Facilitators and barriers to implementing clinical care pathways. BMC Health Serv Res 2010; 10: 1–6. 20584273 10.1186/1472-6963-10-182 PMC2912894

[21] Hynes SM, Field B, Ledgerd R et al Exploring the need for a new UK occupational therapy intervention for people with dementia and family carers: Community Occupational Therapy in Dementia (COTiD). A focus group study. Aging Ment Health 2016; 20: 762–9. 2016/07/02. 25929167 10.1080/13607863.2015.1037243 PMC9122617

[22] Pitkälä KH, Pöysti MM, Laakkonen M et al Effects of the finnish alzheimer disease exercise trial (finalex): A randomized controlled trial. JAMA Intern Med 2013; 173: 894–901. 23589097 10.1001/jamainternmed.2013.359

[23] Pomeroy VM, Warren CM, Honeycombe C et al Mobility and dementia: is physiotherapy treatment during respite care effective? Int J Geriat Psychiatry 1999; 14: 389–97. 10389044

[24] McGilton KS, Davis AM, Naglie G et al Evaluation of patient-centered rehabilitation model targeting older persons with a hip fracture, including those with cognitive impairment. BMC Geriatr 2013; 13: 136. 24330470 10.1186/1471-2318-13-136 PMC4028934

[25] Allan LM, Wheatley A, Flynn E et al Is it feasible to deliver a complex intervention to improve the outcome of falls in people with dementia? A protocol for the DIFRID feasibility study. Pilot Feasibility Stud 2018; 4: 170. 30455976 10.1186/s40814-018-0364-7 PMC6230281

